# The Impact of COVID-19 Concerns on the Mental Health of Older Adults: A Rapid Review

**DOI:** 10.1093/geroni/igab046.2696

**Published:** 2021-12-17

**Authors:** Sophia Perez, Alexandria Nuccio, Ashley Stripling

**Affiliations:** Nova Southeastern University, Fort Lauderdale, Florida, United States

## Abstract

The Coronavirus (COVID-19) Pandemic continues to drastically impact older adults. Despite COVID-19 being linked to increased social isolation and loneliness, more research is needed on the psychological effects associated with older adults’ concerns of the pandemic. The current review explores associations between the COVID-19 Pandemic and older adults’ mental health to increase awareness and understanding. For this rapid review, empirical peer-reviewed source documents were identified through a computerized search using APA PsycInfo and Google Scholar bibliographical databases covering the years 2019 to 2021. The following keywords and combinations were used: “older adults,” “COVID-19,” and “mental health effects.” Relevant exclusion criteria were applied, and all related English-language journal articles were read. 47 articles met inclusion criteria. Eight associated COVID-19 stress with loneliness, anxiety, depression, sleep disturbances, and poor psychological well-being, with three additional articles reporting elevations in women. Three articles revealed mixed findings regarding the impact of age on psychological variables. 13 articles evaluated changes among those with psychological/psychiatric diagnoses, and six explored physical activity and depression. Of the remaining articles, two concentrated on nutrition; seven examined routines, behaviors, and societal or risk perceptions; two evaluated coping mechanisms; and three examined emotional distress changes. Understanding COVID-19’s psychological impact on older adults will take time. This rapid review revealed mixed findings regarding COVID-19 related concerns on older adults’ psychological well-being, with multiple demographic variables uniquely impacting these outcomes. It is imperative that future research explore older adults’ risks and develop interventions related to the psychological impact of COVID-19.

